# Enhanced reporting of deaths among Aboriginal and Torres Strait Islander peoples using linked administrative health datasets

**DOI:** 10.1186/1471-2288-12-91

**Published:** 2012-07-02

**Authors:** Lee K Taylor, Jason Bentley, Jennifer Hunt, Richard Madden, Sybille McKeown, Peter Brandt, Deborah Baker

**Affiliations:** 1 Centre for Epidemiology and Evidence, NSW Ministry of Health, Locked Mail Bag 961, North Sydney, NSW, 2059, Australia; 2 Aboriginal Health and Medical Research Council of New South Wales, PO Box 1565, Strawberry Hills, NSW, 2012, Australia; 3 National Centre for Classification in Health, Faculty of Health Sciences, Cumberland Campus C42, The University of Sydney, PO Box 170, Lidcombe, NSW, 1825, Australia; 4 National Centre for Aboriginal and Torres Strait Islander Statistics, Australian Bureau of Statistics, Locked Bag 10, Belconnen, ACT, 2616, Australia; 5 Demand and Performance Evaluation, NSW Ministry of Health, Locked Mail Bag 961, North Sydney, NSW, 2059, Australia; 6 Monitoring, Evaluation and Research, Cancer Institute NSW, PO Box 41, Alexandria, NSW, 1435, Australia

## Abstract

**Background:**

Aboriginal and Torres Strait Islander peoples are under-reported in administrative health datasets in NSW, Australia. Correct reporting of Aboriginal and Torres Strait Islander peoples is essential to measure the effectiveness of policies and programmes aimed at reducing the health disadvantage experienced by Aboriginal and Torres Strait Islander peoples. This study investigates the potential of record linkage to enhance reporting of deaths among Aboriginal and Torres Strait Islander peoples in NSW, Australia.

**Methods:**

Australian Bureau of Statistics death registration data for 2007 were linked with four population health datasets relating to hospitalisations, emergency department attendances and births. Reporting of deaths was enhanced from linked records using two methods, and effects on patterns of demographic characteristics and mortality indicators were examined.

**Results:**

Reporting of deaths increased by 34.5% using an algorithm based on a weight of evidence of a person being Aboriginal or Torres Strait Islander, and by 56.6% using an approach based on 'at least one report' of a person being Aboriginal or Torres Strait Islander. The increase was relatively greater in older persons and those living in less geographically remote areas. Enhancement resulted in a reduction in the urban-remote differential in median age at death and increases in standardised mortality ratios particularly for chronic conditions.

**Conclusions:**

Record linkage creates a statistical construct that helps to correct under-reporting of deaths and potential bias in mortality statistics for Aboriginal and Torres Strait Islander peoples.

## Background

Improving the health of Aboriginal and Torres Strait Islander peoples is a priority under the Australian Government’s National Partnership Agreement on Closing the Gap on Indigenous Health Outcomes [1]. This Agreement aims to reduce the disadvantage experienced by Aboriginal and Torres Strait Islander peoples with respect to life expectancy, child mortality, access to early childhood education, educational achievement and employment outcomes. Correct reporting of Aboriginal and Torres Strait Islander peoples in health and health-related data collections is essential to measure the effectiveness of policies and programmes aimed at reducing the health disadvantage experienced by Aboriginal and Torres Strait Islander peoples.

New South Wales (NSW) has the largest population and the largest Aboriginal and Torres Strait Islander population of all Australian States and Territories; comprising 7.2 million people (32% of the Australian population), and about 155,700 people (30% of the Australian Aboriginal and Torres Strait Islander population) respectively [2].

As Aboriginal or Torres Strait Islander people are not always correctly reported in death registrations on which the Australian Bureau of Statistics (ABS) death data are based, the number of deaths among Aboriginal and Torres Strait Islander peoples is under-reported. Using a record linkage method (linking Census to death registrations data), the ABS estimated that the rate of reporting of deaths among Aboriginal and Torres Strait Islander peoples was 76% in 2006–07 [3].

As the Census is carried out every five years, a mechanism is needed to correct the under-reporting of deaths among Aboriginal and Torres Strait Islander peoples on an annual basis. Record linkage of routinely collected health data with ABS death data provides a possible method to increase reporting of deaths among Aboriginal and Torres Strait Islander peoples. Information on Aboriginal and Torres Strait Islander peoples is drawn from all linked records and used to ‘enhance’ information on the ABS death data.

Record linkage has been used previously to enhance reporting of indigenous peoples on mortality data in Australia, New Zealand and Canada using various approaches [3-8]. Methods have included reporting a person as indigenous where: any linked record indicates the person is indigenous; a person is reported as indigenous on at least 50% of linked records; a person is reported as indigenous on at least 2 linked records and/or records from at least 2 hospitals; or where information on whether the person is indigenous is missing from the dataset of interest and a linked record reported that they are indigenous. Many of these previous approaches do not take into account the possibility of incorrect enhancement due to occasional incorrect links or data entry errors resulting in incorrect reports of a non-indigenous person as indigenous in the source datasets. While the overall rate of such problems may be low, where linked datasets are very large, and indigenous populations are relatively small, a low rate of incorrect links or data entry errors could make a substantial difference to the number of deaths reported after enhancement.

This study estimates the level of increased reporting of deaths among Aboriginal and Torres Strait Islander peoples in NSW on the ABS death data that is achieved by using linked records from a range of health and health-related datasets, and the impact on mortality rates. Baseline reporting of deaths on the ABS data is compared with two methods of enhancement: first, where there is any linked record that indicates a person is Aboriginal or Torres Strait Islander; and second, an algorithm that uses a weight of evidence to report a person as Aboriginal or Torres Strait Islander.

## Methods

Ethical approval was obtained from the NSW Population and Health Services Research Ethics Committee and the Aboriginal Health and Medical Research Council (AH&MRC) Ethics Committee. A Reference Group of community members nominated by the AH&MRC provided advice on issues relevant to Aboriginal and Torres Strait Islander peoples in NSW and on interpretation of results.

### Data sources

ABS compiles and processes death registration data collected by Australian state and territory Registrars of Births, Deaths and Marriages (RBDM). Records of birth registrations were obtained from the NSW RBDM. Birth and death registration data are based on the year of registration of the birth or death. The following data were obtained from the NSW Ministry of Health:

· The NSW Admitted Patient Data (APD) covers demographic and episode related data for every inpatient that is admitted to any public, private, and repatriation hospital, private day procedure centre, or public nursing home in NSW. APD data are based on the year of hospital separation.

· The NSW Emergency Department Data Collection (EDDC) covers demographic and emergency treatment related data for every person that presents to major public emergency departments in NSW. EDDC data are based on the year of emergency department attendance.

· The NSW Perinatal Data Collection (PDC) covers demographic and birth related data for every birth in NSW in public and private hospitals and homebirths and includes information on mothers and infants. PDC data are based on the year of baby’s birth.

### Record linkage and dataset preparation

The APD (1 July 2000 – 31 December 2007), EDDC (1 January 2005 – 31 December 2007), PDC (1 January 2000 – 31 December 2007), RBDM birth registration data (1 January 2000 – 31 December 2007) and ABS death data (deaths registered in NSW in 2007) were linked by the Centre for Health Record Linkage (CHeReL) [9]. The CHeReL uses a best practice approach in privacy preserving record linkage [10] and the open source probabilistic record linkage software ChoiceMaker [11]. The CHeReL used the following information on the APD, PDC, and RBDM birth registration datasets to probabilistically link records for the same person: full name, mother’s name (in the case of a birth), address, sex, date of birth, country of birth, hospital code, medical record number, hospital dates of admission and discharge, hospital transferred to, hospital transferred from, date of death, and date of emergency department attendance. ChoiceMaker uses ‘blocking’ and ‘scoring’ to identify definite and possible matches. During blocking ChoiceMaker searches the target datasets for records that are possible matches to each other. There are two types of blocking: exact blocking requires records to have the same set of valid fields and the same values for these fields; automated blocking builds a set of conditions to find as many records as possible that potentially match each other. Scoring employs a combination of a probabilistic decision, computed using a machine learning technique, and absolute rules, including upper and lower probability cut-offs, to determine the final decision as to whether each match denotes or possibly denotes the same person.

ABS death data were deterministically linked to RBDM death registration records using the death registration number. Information on whether a person was Aboriginal or Torres Strait Islander was not used for record linkage. For the entire linked dataset the CHeReL reported the linkage quality as less than 5/1,000 missed links and 4/1,000 false positive links.

The final analysis dataset comprised 46 139 ABS death records linked to 648 746 records from population datasets: APD *n* = 511 949, EDDC *n* = 135 657, RBDM birth registration records (mothers) *n* = 194, RBDM birth registration records (infants) *n* = 332, PDC records (mothers) *n* = 211, and PDC records (infants) *n* = 403. There was at least one linked record for 44 328 (96.1%) deaths.

### Data analysis

Due to the small numbers of deaths among Torres Strait Islander people in NSW, deaths among Aboriginal and Torres Strait Islander peoples were considered as a group for the purpose of the analysis.

For babies, if the mother was recorded as Aboriginal or Torres Strait Islander on the PDC, the baby was recorded as Aboriginal or Torres Strait Islander. Similarly, for the RBDM birth registrations, if the mother or father were recorded as Aboriginal or Torres Strait Islander, then the baby was recorded as Aboriginal or Torres Strait Islander.

Persons reported as Aboriginal or Torres Strait Islander on the ABS death data were accepted as reported. We considered various approaches to assessing the weight of evidence from linked records where a person was reported as non-Aboriginal or Torres Strait Islander on the ABS death record or where this information was missing. Previous work using linked death and hospital records found that an algorithm relying on the number of linked records and number of hospitals reporting a person as Aboriginal or Torres Strait Islander resulted in a higher number of reported deaths than an algorithm relying on a proportion of linked records and/or hospitals [8]. From this we developed the concept of a ‘unit of information’, which relies on information about whether a person is Aboriginal or Torres Strait Islander being collected independently for each data collection and each health service encounter. For this study, we defined a ‘unit of information’ as information on an Aboriginal or Torres Strait Islander person obtained from one of: a RBDM birth registration record, a PDC record, an EDDC record, an ABS death record or a record from the APD representing a hospital stay.

The number of deaths among Aboriginal and Torres Strait Islander peoples that were recorded in ABS death data was compared to the number of deaths ascertained by the following two enhancement methods:

1. If at least one linked record reported the person as Aboriginal or Torres Strait Islander then the death was considered to be of an Aboriginal or Torres Strait Islander person.

2. According to the following algorithm:

a) a death reported for an Aboriginal or Torres Strait Islander person on the ABS death data was accepted as reported;

b) for remaining deaths:

i) if the person had 3 or more linked units of information, then the death was considered to be of an Aboriginal or Torres Strait Islander person where at least 2 linked units of information reported the person as Aboriginal or Torres Strait Islander; or

ii) if the person had 1 or 2 linked units of information the death was considered to be of an Aboriginal or Torres Strait Islander person where at least 1 linked unit of information reported the person as Aboriginal or Torres Strait Islander.

We examined unenhanced and enhanced counts of deaths by age, sex, geographic remoteness and cause of death. Geographic remoteness was measured using the Accessibility/Remoteness Index of Australia (ARIA+) [12]; 1.2% of death records could not be assigned an ARIA + code. We examined median age at death, and indirectly standardised mortality ratios (SMRs) for cardiovascular diseases (ICD-10 [13]: I00-I99), cancer (ICD-10: C00-C97) and external causes (ICD-10: V01-Y98). SMRs were calculated as follows: standard death rates by five year age group were obtained using ABS Australian death data for 2006 and the ABS estimated resident Australian population for the Census year 2006 [14]; these rates were applied to the ABS estimated Aboriginal and Torres Strait Islander population NSW 2007 [2] by sex and five year age group and summed to obtain the expected number of Aboriginal and Torres Strait Islander deaths; finally, the ratios of observed number of deaths for the three groups ‘as reported’, the algorithm and ‘at least one report’ were compared to the expected number of deaths to give SMRs for the three groups. Exact confidence intervals were calculated for the SMRs using the Gamma distribution. Analyses were carried out using SAS 9.2 [15].

## Results

There were 580 ABS records of deaths among Aboriginal and Torres Strait Islander peoples that were registered in NSW in 2007. This represents the minimum number of reported deaths and provides a baseline for comparison. After record linkage, enhancement resulted in 780 reported deaths using the algorithm and 908 reported deaths using ‘at least one linked record’ where the person was reported as Aboriginal or Torres Strait Islander (Table 1).

**Table 1:** Deaths among Aboriginal and Torres Strait Islander peoples by method of reporting and demographic characteristics, New South Wales Australia 2007

**Demographic characteristics**		**Reporting method**				
		**As reported**	**Enhanced reporting**			
			**Algorithm**^**a**^		**At least 1 report**	
		**No.**	**No.**	**Increase%**^**b**^	**No.**	**Increase%**^**b**^
Age (years)	0	38	45	18.4	50	31.6
	1- 4	6	7	16.7	8	33.3
	5 - 9	1	1	0.0	1	0.0
	10 - 14	2	4	100.0	5	150.0
	15 - 19	3	4	33.3	4	33.3
	20 - 24	6	9	50.0	9	50.0
	25 - 29	7	11	57.1	11	57.1
	30 - 34	16	21	31.3	22	37.5
	35 - 39	20	25	25.0	30	50.0
	40 - 44	36	46	27.8	49	36.1
	45 - 49	50	62	24.0	66	32.0
	50 - 54	47	60	27.7	64	36.2
	55 - 59	45	57	26.7	64	42.2
	60 - 64	65	80	23.1	89	36.9
	65 - 69	59	78	32.2	90	52.5
	70 - 74	55	73	32.7	83	50.9
	75 - 79	38	58	52.6	77	102.6
	80 - 84	29	47	62.1	65	124.1
	85 +	57	92	61.4	121	112.3
Sex	Male	311	420	35.0	486	56.3
	Female	269	360	33.8	422	56.9
Geographic remoteness (ARIA+)^c^	Major Cities	171	247	44.4	293	71.3
	Inner Regional	190	255	34.2	304	60.0
	Outer Regional	137	178	29.9	203	48.2
	Remote	47	55	17.0	60	27.7
	Very Remote	23	26	13.0	28	21.7
Cause of death	Cancer^**d**^	128	188	46.9	227	77.3
	Cardiovascular diseases^**e**^	181	234	29.3	273	50.8
	External causes^**f**^	56	73	30.4	85	51.8
Total^g^		580	780	34.5	908	56.6

Data source: Australian Bureau of Statistics death data linked with records of the NSW Admitted Patient Data, NSW Emergency Department Data Collection, NSW Perinatal Data Collection and Registry of Births, Deaths and Marriages birth registration data.

^**a**^For 3 or more linked units of information 2 are required to report an individual as Aboriginal or Torres Strait Islander, otherwise 1 is sufficient.

^**b**^Difference between the number of enhanced deaths and the “As-reported” deaths as a percentage of the “As-reported” number of deaths.

^**c**^Accessibility/Remoteness Index of Australia—ARIA Plus ^12^.

^**d**^Cancer cause of death codes ICD-10: C00-C97.

^**e**^Cardiovascular diseases cause of death codes ICD-10: I00-I99.

^**f**^External causes of death codes ICD-10: V01-Y98.

^**g**^Total includes records with missing information on demographic characteristics.

After enhancement, apart from those less than one year of age, there was little increase in the numbers of reported deaths among Aboriginal and Torres Strait Islander children and young people, while increasing numbers of reported deaths were observed with increasing age. After enhancement, there was also a greater proportional increase in reported deaths among those aged 75 years and over, compared to younger adult age groups, while the proportional increase in reported deaths among children and young people was quite variable between age groups.

Each enhancement method produced similar rates of increased reporting for both males and females. For geographic remoteness, the increase in number of reported deaths was greatest in the major cities, with enhancement rates decreasing with increasing remoteness. While deaths due to cardiovascular diseases were most common of the three groups, rates of enhancement among persons who died of cancer were substantially higher than those of persons who died of cardiovascular diseases.

The median age at death based on unadjusted ABS death data was higher for males and females combined living in remote and very remote areas compared to more urban areas (Table 2). After enhancement there was an increase in the median age at death among males and females combined for those living in major cities, with small variations in other remoteness groups. The effect of enhancement was to reduce the urban-remote differential in median age at death.

**Table 2:** Median age at death for Aboriginal and Torres Strait Islander peoples by method of reporting, sex, cause of death, and geographic remoteness, New South Wales Australia 2007

**Cause of death – geographic remoteness**		**Reporting method**								
		**As reported**			**Enhanced reporting**					
					**Algorithm**^**a**^			**At least 1 report**		
		**Male**	**Female**	**Total**	**Male**	**Female**	**Total**	**Male**	**Female**	**Total**
Cause of death	Cancer^**b**^	64.4	63.6	64.3	64.6	62.5	63.8	65.2	64.4	65.0
	Cardiovascular diseases^**c**^	61.1	72.0	65.9	64.8	73.5	69.1	66.0	76.5	71.2
	External causes^**d**^	36.8	40.4	39.5	39.9	39.7	39.7	38.0	40.4	39.7
Geographic remoteness (ARIA+) ^**e**^	Major Cities	59.5	66.1	61.7	61.0	66.3	62.8	62.4	67.1	64.9
	Inner Regional	62.5	61.9	62.1	61.9	65.2	63.0	62.9	68.8	65.1
	Outer Regional	56.0	65.7	59.9	57.8	63.4	60.5	59.6	65.0	61.1
	Remote	60.0	58.8	59.9	61.2	60.6	61.2	63.2	63.0	63.2
	Very Remote	63.2	65.0	64.4	62.9	65.0	63.9	63.2	69.1	64.7
Total ^**f**^		59.5	63.5	61.1	60.8	65.1	62.6	62.1	67.0	63.9

Data source: Australian Bureau of Statistics death data linked with records of the NSW Admitted Patient Data, NSW Emergency Department Data Collection, NSW Perinatal Data Collection and Registry of Births, Deaths and Marriages birth registration data.

^**a**^For 3 or more linked units of information 2 are required to report an individual as Aboriginal or Torres Strait Islander, otherwise 1 is sufficient.

^**b**^Cancer cause of death codes ICD-10: C00-C97.

^**c**^Cardiovascular diseases cause of death codes ICD-10: I00-I99.

^**d**^External causes of death codes ICD-10: V01-Y98

^**e**^Accessibility/Remoteness Index of Australia—ARIA Plus ^12^.

^**f**^Total includes records with missing information ARIA+ or cause of death.

The median age at death for cardiovascular diseases rose by 3.2 years after enhancement with the algorithm, and 5.3 years after enhancement with ‘at least one report’; median ages at death for males and females followed a similar pattern. For cancer and external causes of death, the median age at death varied little with the two enhancement methods.

Enhancement resulted in higher standardised mortality ratios (SMRs) for all causes of death examined and for both sexes (Table 3). As expected, enhancement based on ‘at least one report’ resulted in higher standardised mortality ratios compared with enhancement based on the algorithm. For males the largest absolute increase in SMR was for cancer followed by cardiovascular diseases and then external cause mortality; for females the largest absolute increase was for cardiovascular diseases, followed by cancer and then external causes.

**Table 3:** Standardised mortality ratios for Aboriginal and Torres Strait Islander peoples by method of reporting, sex and cause of death, New South Wales Australia 2007

**Cause of death**	**Method of enhancement**	**Males**				**Females**				**Total**			
		**SMR**	**95% CI**			**SMR**	**95% CI**			**SMR**	**95% CI**		
Cancer^**a**^	As reported	152.3	119.6	-	191.2	130.3	97.9	-	170.1	141.1	117.7	-	167.8
	Algorithm^**e**^	223.3	182.3	-	268.4	193.1	153.1	-	240.3	207.2	178.7	-	239.1
	At least one report	275.8	231.1	-	326.6	224.5	181.2	-	275.0	250.2	218.7	-	285.0
Cardiovascular diseases^**b**^	As reported	261.6	212.8	-	318.1	252.9	200.8	-	314.3	254.7	219.0	-	294.6
	Algorithm^**e**^	329.6	274.6	-	392.4	337.2	276.6	-	407.1	329.3	288.5	-	374.3
	At least one report	368.8	310.5	-	435.0	412.1	344.8	-	488.7	384.2	340.0	-	432.6
External causes^**c**^	As reported	126.8	88.3	-	176.3	205.8	127.4	-	314.6	146.6	110.7	-	190.4
	Algorithm^**e**^	173.9	128.2	-	230.6	245.0	158.5	-	361.6	191.1	149.8	-	240.3
	At least one report	195.6	147.0	-	255.3	303.8	206.4	-	431.2	222.5	177.7	-	275.2
Total	As reported	190.6	170.0	-	213.0	213.0	188.3	-	240.1	198.7	182.9	-	215.6
	Algorithm^**e**^	257.4	233.4	-	283.3	285.1	256.4	-	316.1	267.3	248.8	-	286.7
	At least one report	297.9	272.0	-	325.6	334.2	303.0	-	367.6	311.1	291.2	-	332.1

Data sources: Australian Bureau of Statistics (ABS) death registration data for NSW linked with records of the NSW Admitted Patient Data, NSW Emergency Department Data Collection, NSW Perinatal Data Collection and Registry of Births, Deaths and Marriages birth registration data; ABS Aboriginal and Torres Strait Islander estimated resident population NSW 2007^2^; and standard death rates were obtained using ABS Australian death data 2006 and the estimated resident Australian population 2006 ^14^.

*CI* Confidence interval.

^**a**^Cancer cause of death codes ICD-10: C00-C97.

^**b**^Cardiovascular diseases cause of death codes ICD-10: I00-I99.

^**c**^External causes of death codes ICD-10: V01-Y98.

^**e**^For 3 or more linked units of information 2 are required to report an individual as Aboriginal or Torres Strait Islander, otherwise 1 is sufficient.

## Discussion

Enhancement of reporting of deaths among Aboriginal and Torres Strait Islander peoples using record linkage with a range of population datasets resulted in a substantial increase in the number of reported deaths. Compared to the baseline reporting of 580 deaths in 2007, an algorithm based on assessing the weight of evidence of a person being Aboriginal or Torres Strait Islander increased reporting by an additional 200 (34.5%) deaths. Enhancement using ‘at least one report’ of a person being Aboriginal or Torres Strait Islander increased reporting by an additional 328 (56.6%) deaths. The level of reporting of deaths among Aboriginal and Torres Strait Islander peoples in NSW in the ABS death data is therefore estimated at 74.4% based on enhancement with the algorithm or 63.9% based on ‘at least 1 report’.

In relation to age, the greatest enhancement in reporting of deaths was found in older people. As hospital records comprised 78.9% of the linked records and hospitalisation is more common among older people, there was a greater opportunity to enhance reporting of deaths among older Aboriginal or Torres Strait Islander people compared to younger people. There was also greater enhancement of reported deaths for those with chronic conditions, which are likely to generate many hospital records, compared to acute conditions. Enhanced reporting of deaths resulted in increases in SMRs, with a greater proportional increase in SMRs for cancer and cardiovascular diseases compared to external causes of death.

It is not known whether the observed differential enhancement of number of deaths by age resulted in a biased age distribution in the enhanced dataset, or served to correct a reporting bias in the original dataset. While not examined as part of this project, the observed differential enhancement of death data by age would be expected to change estimates of life expectancy for Aboriginal and Torres Strait Islander peoples. Further research is needed to ascertain whether the age distribution in the enhanced dataset is a true reflection of the age distribution of Aboriginal and Torres Strait Islander peoples who died. For example, linkage of the enhanced dataset with a sample of records from a dataset that is known to have reliable reporting of Aboriginal and Torres Strait Islander peoples, such as records from Aboriginal community controlled health services, could be used to explore this.

For geographic remoteness, enhanced reporting of deaths was associated with decreasing geographic remoteness of residence from remote areas to major cities, resulting in a reduction in the urban-remote differential in median age at death. The percentage increase in number of deaths resulting from enhancement was similar for males and females.

The level of reporting of deaths among Aboriginal and Torres Strait Islander peoples in NSW on ABS death data based on enhancement with the algorithm (74.4%) is similar to that found by ABS for NSW deaths in a eleven-month period in 2006 and 2007 using linked death and Census records (76.3%) [3]. The pattern of increased enhancement for older persons and non-remote regions observed in this study was also observed by Briffa et al [6] in Western Australia using the ‘at least one report’ approach.

There is some advantage in using administrative health datasets for linkage as these are available on a continuing basis, whereas Census data are available every 5 years. In Australia, Census data are available for linkage only for a short time after the Census as personal identifiers are removed once the dataset is finalised.

In considering whether to use an approach based on an algorithm that uses the weight of evidence for whether a person is indigenous or an approach based on ‘at least one report’, the likelihood of misclassification of an indigenous person as non-indigenous or vice-versa should be taken into account. A national survey estimated the level of correct reporting of Aboriginal and Torres Strait Islander peoples on NSW public hospital admitted patient data in 2007 to be 88% [16], while an analysis of linked records estimated the level of correct reporting of Aboriginal and Torres Strait Islander peoples on the PDC to be 68.0% [17]. There is no information on the quality of reporting of Aboriginal and Torres Strait Islander peoples on the EDDC or RBDM birth registration data. In terms of misclassification of a non-indigenous person as indigenous, incorrect links or incorrect reporting on the source record should be considered. In order to create the observed difference of 128 deaths between the two enhancement methods in this study, a misclassification rate of about 1.9 per 10,000 linked records would be required. Thus, an extremely low misclassification rate in a large linked dataset can make a substantial difference to the number of reported deaths among indigenous peoples when an ‘at least one report’ method of enhancement is used. We suggest that, for enhancement methods using administrative health datasets, the preference should be towards an algorithm that incorporates a weight of evidence. In this study the number of deaths reported among Aboriginal and Torres Strait Islander peoples on the ABS death data is relatively small (*n* = 580), the chance of incorrect reporting of a non-Aboriginal or Torres Strait Islander person as Aboriginal or Torres Strait Islander is also likely to be small, and we suggest that this information be accepted as reported.

It is likely that some deaths of Aboriginal or Torres Strait Islander people are not included in the enhanced counts. There were no linked records for 3.9% of ABS death records. It was not possible to attempt to enhance reporting of deaths for those Aboriginal or Torres Strait Islander people who did not have a relevant health service encounter, or given birth or been born, in the period covered by the study. It is also possible that some Aboriginal or Torres Strait Islander people were not reported as Aboriginal or Torres Strait Islander on the ABS death record or on any of their linked records. It is therefore likely that the count of deaths based on enhancement with the algorithm still represents an under-estimate of the true number of deaths.

Enhancement of reporting of deaths using record linkage does not define whether a person is indigenous. Rather, record linkage results in a statistical construct created for the purposes of planning and research. It provides a mechanism to help reduce the under-reporting of deaths among indigenous peoples in official statistics, and allows adjustment of historical data to obtain improved estimates of the mortality experience of indigenous peoples. Importantly in this study, record linkage resulted in correction of some of the bias in mortality measures resulting from relative under-reporting of Aboriginal and Torres Strait Islander peoples resident in major cities and less remote geographic areas.

We chose to carry out enhancement using all available linked records. A smaller number of years of linked data could have been used, and would have resulted in a different number of reported deaths. Any statistical construct will depend on the purpose for which the data are intended to be used. For example, if the purpose was to examine trends in mortality among over several years, the range of datasets and the years of linked data used should be consistent for each year included in such a study.

Various approaches are possible for algorithms incorporating a weight of evidence, such as a requirement that a certain percentage (e.g. 50%, 75% or 90%) of linked records report that the person is indigenous. Algorithms based on a proportion of records reporting a person as indigenous require a greater weight of evidence than the algorithm used in this study, and would result in a relatively smaller increase in the number of deaths reported as a result of the enhancement. We believe that enhanced reporting of deaths using the algorithm developed in this study provides a balance between achieving a reasonable weight of evidence that a person is indigenous, and maximising the number of additional deaths found through the enhancement.

It would also be possible to develop algorithms where different data collections carry different weights of evidence. For example, linkage could include records from health services that are dedicated to providing services to indigenous people, and these records could provide a greater weight of evidence that a person is indigenous than records collected as part of universal health services. Factors that should be taken into account in determining which datasets should be linked for enhancement purposes include: previous validations studies, representativeness of the community, and the extent to which information in each dataset is collected independently.

Finally, while record linkage provides a mechanism to deal with the issues of under-reporting of deaths among indigenous peoples and to help correct reporting in historical data, it is not a replacement for continued efforts to increase reporting of indigenous peoples on administrative health data collections and death registrations.

## Conclusions

Record linkage provides a mechanism to help reduce the under-reporting of deaths among indigenous peoples in official statistics, and allows adjustment of historical data to obtain improved estimates of the mortality experience of indigenous peoples. In this study, record linkage enabled correction of some of the bias in mortality measures resulting from relative under-reporting of Aboriginal and Torres Strait Islander peoples resident in major cities and less remote geographic areas.

## Competing interests

The authors declare that they have no competing interests.

## Authors’ contributions

LKT and JB conceptualised the study. All authors contributed to the development of the methods. JB carried out the data analysis. JB and LT drafted the manuscript. All authors participated in the revision of the manuscript, and read and approved the final manuscript.

## Pre-publication history

The pre-publication history for this paper can be accessed here:

http://www.biomedcentral.com/1471-2288/12/91/prepub
